# The influence of protein corona on Graphene Oxide: implications for biomedical theranostics

**DOI:** 10.1186/s12951-023-02030-x

**Published:** 2023-08-11

**Authors:** Erica Quagliarini, Daniela Pozzi, Francesco Cardarelli, Giulio Caracciolo

**Affiliations:** 1 NanoDelivery Lab, Department of Molecular Medicine, Sapienza University of Rome, Viale Regina Elena 291, 00161 Rome, Italy; 2 NEST Laboratory, Scuola Normale Superiore, Piazza San Silvestro 12, 56127 Pisa, Italy

**Keywords:** Graphene oxide, Protein corona, Nanotechnology, Theranostics

## Abstract

Graphene-based nanomaterials have attracted significant attention in the field of nanomedicine due to their unique atomic arrangement which allows for manifold applications. However, their inherent high hydrophobicity poses challenges in biological systems, thereby limiting their usage in biomedical areas. To address this limitation, one approach involves introducing oxygen functional groups on graphene surfaces, resulting in the formation of graphene oxide (GO). This modification enables improved dispersion, enhanced stability, reduced toxicity, and tunable surface properties. In this review, we aim to explore the interactions between GO and the biological fluids in the context of theranostics, shedding light on the formation of the “protein corona” (PC) i.e., the protein-enriched layer that formed around nanosystems when exposed to blood. The presence of the PC alters the surface properties and biological identity of GO, thus influencing its behavior and performance in various applications. By investigating this phenomenon, we gain insights into the bio-nano interactions that occur and their biological implications for different intents such as nucleic acid and drug delivery, active cell targeting, and modulation of cell signalling pathways. Additionally, we discuss diagnostic applications utilizing biocoronated GO and personalized PC analysis, with a particular focus on the detection of cancer biomarkers. By exploring these cutting-edge advancements, this comprehensive review provides valuable insights into the rapidly evolving field of GO-based nanomedicine for theranostic applications.

**Graphical Abstract:**

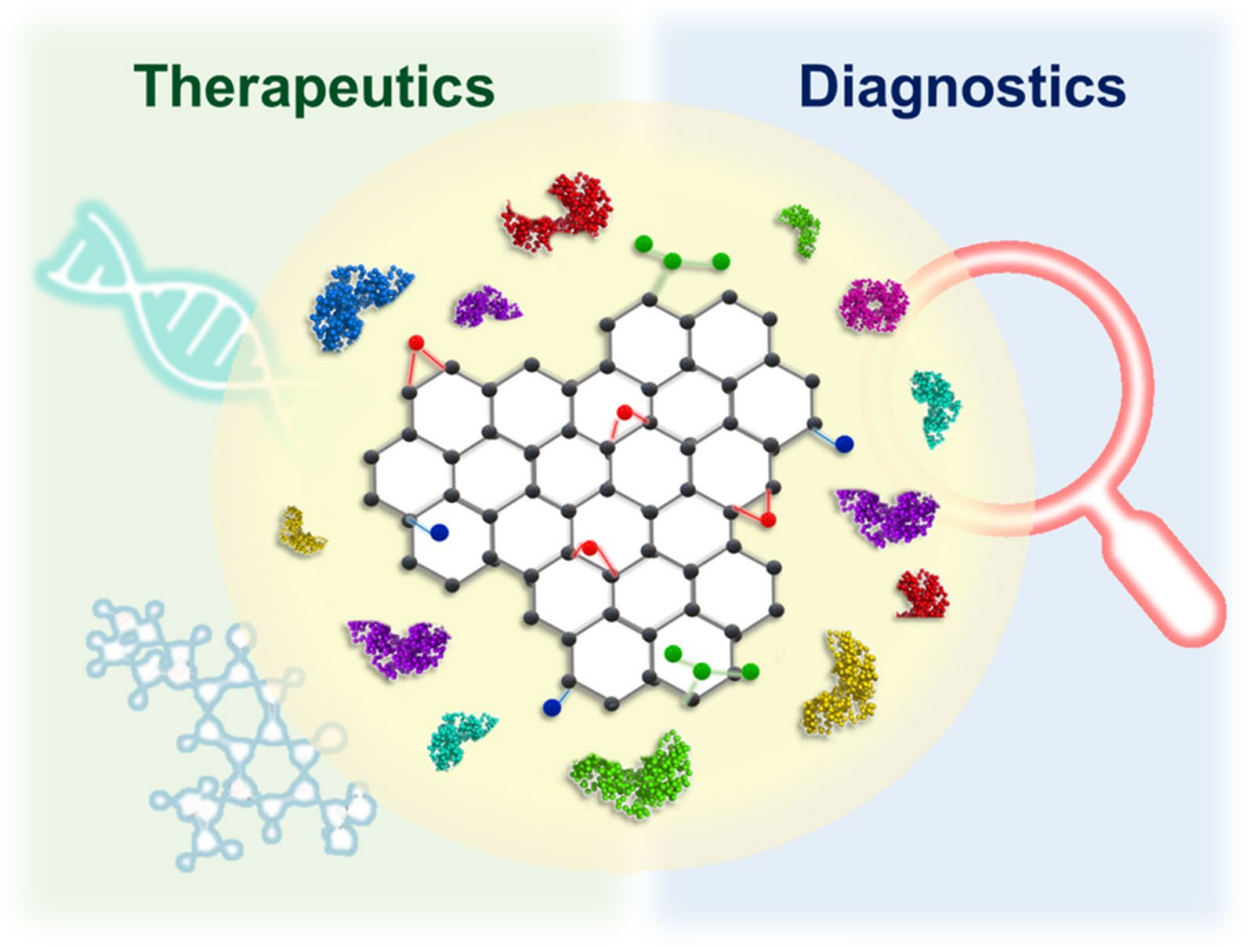

## Introduction

**Fig. 1:**
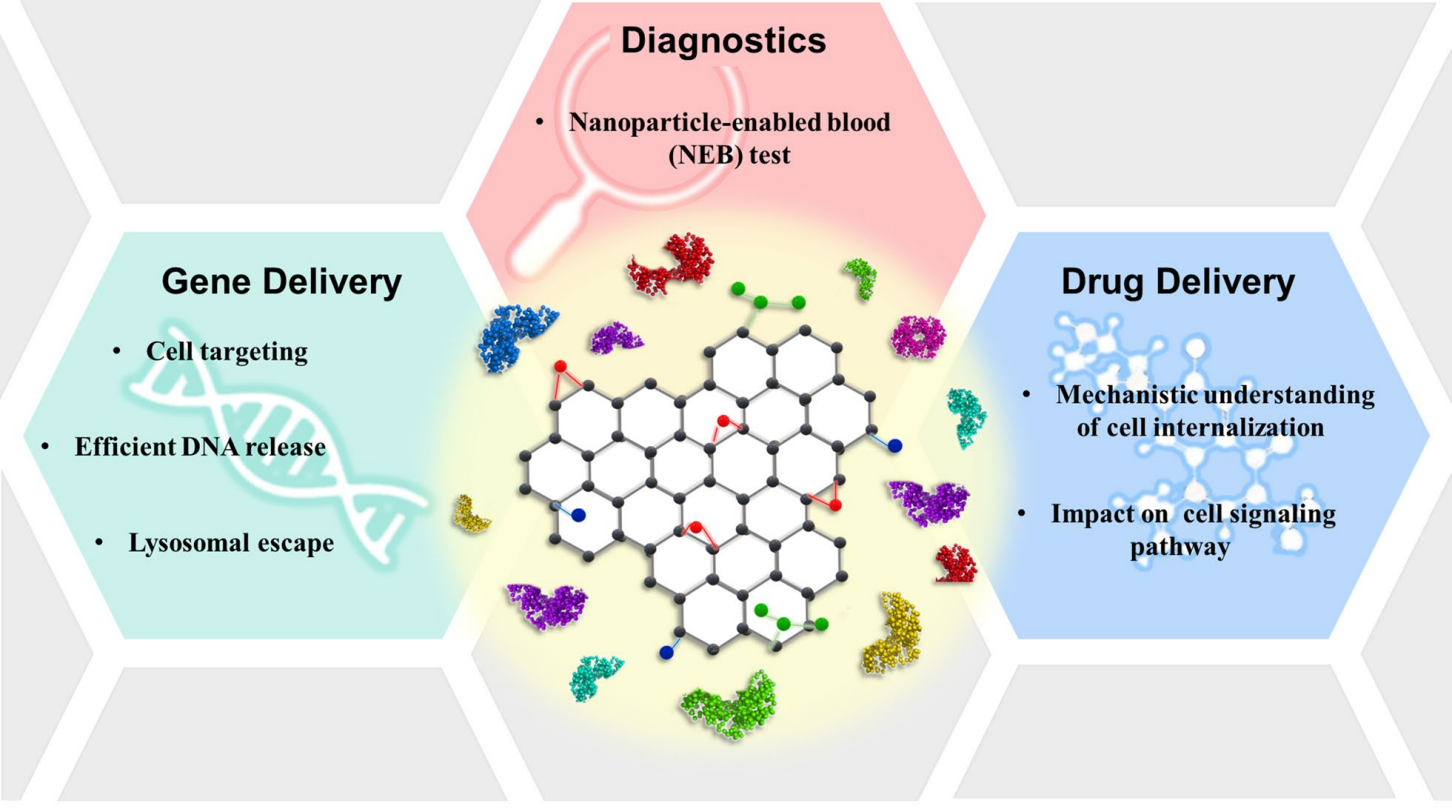
Application of biocoronated Graphene Oxide in gene delivery, drug delivery and diagnostics

Over the years, 2D nanomaterials have provided fertile ground for the emergence of high-performance technologies in nanomedicine [1]. Among 2Ds, graphene-based ones have been largely exploited in the field due to their structural characteristics deriving from the unique atomic arrangement. The manifold applications of these materials (including Graphene oxide (GO), reduced graphene oxide (rGO), and graphene quantum dots) have engendered in the biological arena, including but not limited to nanocarrier fabrication [2], drug delivery [3], cancer therapy [4], and tissue engineering [5]. However, due to their high hydrophobicity, most of these materials demonstrated high toxicity within biological systems, thus limiting their use [6, 7]. Despite the interesting properties, the use of graphene flakes in biological environments without any modification proved to be quite challenging. The use of GO overcomes this issue. In fact, the presence of oxygen functional groups on GO surface guarantees enhanced dispersion in water solution and easier functionalization with biological molecules, finally providing a material with improved stability, reduced toxicity, and tunable surface properties [8, 9]. In addition, the presence of oxidated functional groups confers to the nanomaterial a high affinity towards biomolecules, such as DNA or proteins, allowing easy functionalization for targeting intent or biomarkers detection [10–12]. All these aspects enable new and promising opportunities in biomedical research, particularly in the domain of cancer investigation. Despite the abundant progress, there are still primary concerns and urgent challenges that need to be addressed before the clinical application of GO. One major concern is the toxicity and biosafety of GO, as nanomaterials require rigorous evaluation before clinical approval. Although numerous studies have investigated the in vitro and in vivo toxicity of GO and its derivatives, there are still uncertainties regarding their clinical application. To facilitate the clinical translation of GO, factors such as stability in physiological conditions, interaction with cells, cellular response, uptake mechanism, biodistribution, transformation and metabolism in vivo need to be carefully considered. Size and surface properties significantly influence the toxicity of nanomaterials, and researchers can tailor suitable GO-based nanomaterials by controlling their size, oxidation degree, and surface modification by biocompatible agents. Surface engineering of GO is crucial to empower nanomaterials with superior properties for biomedical applications, such as hydrophilicity, stability, affinity, and biodegradability. The covalent or non-covalent modification enables the decoration of GO surface with various agents, including PEG, PEI, PLA, PLL, and RGD [11, 13]. However, some surface agents are not biodegradable in vivo and may pose risks, while others may be unstable in physiological environments [14]. Achieving a suitable conjugation ratio while maintaining a balance between the defects and desired biomedical functions are both critical factors for a successful application of GO. The size of GO is important for efficient passive tumor targeting through the enhanced permeability and retention (EPR) effect, considering the limitations of endocytosis for large-sized nanomaterials and rapid clearing for ultra-small-sized nanomaterials. Tumor targeting performance plays a key role in tumor diagnosis and therapy, where agents need to be efficiently delivered and retained in the tumor tissue. Specific active tumor targeting can be achieved by conjugating targeting agents to GO and concomitantly exploiting the overexpression of receptors on tumor cell membranes. Moreover, leveraging endogenous and exogenous stimuli to achieve smart regulation of GO-based nanoplatforms within tumors is essential for precise diagnosis and therapy. The rapid development of personalized medicine necessitates the integration of multiple functions within a single nanoparticle. Building on the foundation of GO, functional agents can be used to provide multimodal functions. However, current strategies face challenges such as complex design, laborious synthesis, low integration efficiency, lack of synergistic functions, and uncertain biological responses. Designers must carefully consider the rational combination of necessary functions on GO, aligning with the biological demands of clinical practice. The application of nanotechnology in cancer research allowed to tackle many limitations of conventional therapeutic or diagnostic technologies [15, 16]. Notably, emerging studies on the interaction between nanomaterials and biological systems have provided novel insights and perspectives for the design of nanomedicine. In a physiological environment, nanomaterials encounter various fluids, including blood. Blood counts with a protein concentration of about 60–80 mg/ml with 3700 types of proteins identified to date, including high-abundance proteins such as human serum albumin (HSA) and transferrin, stroke proteins such as receptor ligands and cytokines, and low-abundance proteins such as those derived from tissue or cell secretions [15]. Given their high abundance, proteins inevitably attach to the surface of nanomedicines leading to the formation of a “protein corona” (PC) [17]. PC alters the surface conformation and physicochemical properties of the pristine nanomaterials (i.e., their “synthetic identity”), thus, shaping a new “biological identity” that ultimately leads to a specific physiological response [18, 19]. Exploring the bionano interactions with the biological milieu has therefore emerged as the missing link between benchtop discoveries and the clinical applicability of nanomedicines. The formation of PC on graphene-based materials has been the subject of recent studies [20, 21]. For instance, Liu et al. studied the influence of HSA on GO surface at different pH values and demonstrated that the attachment of GO to a model cell membrane was reduced in the presence of HSA corona [22]. In another study, a thorough examination was conducted to understand the impact of GO nanosheets on cells when exposed to various levels of fetal bovine serum (FBS). When FBS concentration was low (1%), human cells exhibited sensitivity towards GO and demonstrated cytotoxicity that varied with FBS concentration. Surprisingly, the cytotoxic effect of GO was significantly reduced when the FBS concentration was increased to 10%, which is typically used in cell culture media [23]. Compared to the numerous review papers already existing in the literature, this work aims at discussing the role of the bio-nano interactions between GO and plasma proteins in the theranostics field. To this end, we will first detail the use of GO for the delivery of nucleic acids and drugs. Particularly, we will show how the physicochemical and functional properties of GO are modified by the adsorption of a PC allowing for active cell targeting, and efficient cargo release but also alteration of cell receptor interaction and cell signalling pathways. Lastly, a comprehensive exploration of the diagnostic applications of biocoronated GO will be provided, emphasizing the emerging concept of personalized protein corona (PC). In this context, the focus will be on the analysis of PC derived from clinically relevant biological fluids, showcasing the potential and relevance of this approach. Notably, we will present the possibility of cancer detection through an outstanding analytical technology that exploits the personalized PC of GO as a sensor for biomarker detection. With this review, our aim is to offer readers a comprehensive overview of the latest and most noteworthy advancements in the realm of biocoronated GO applications. By doing so, we strive to provide a refreshed perspective on the significant discoveries in this field (Fig. 1).

## Exploring the evolution of Graphene Oxide-Based gene vectors: from synthetic constructs to Biological entities

**Fig. 2:**
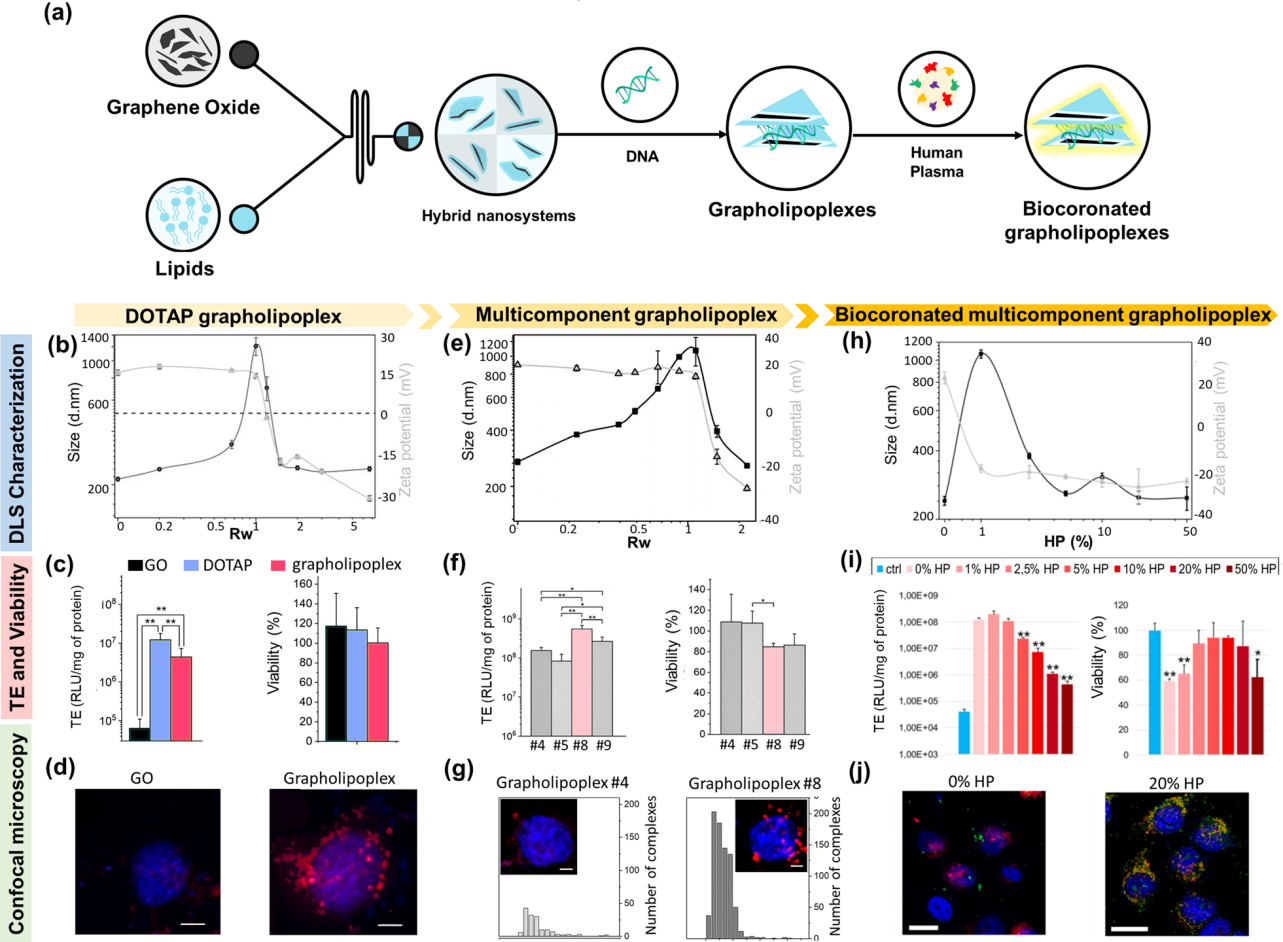
Synthetic evolution of hybrid gene delivery systems made of GO nano-sheets surface-functionalized with lipids described by a multi-step validation approach.** a** Sketch of the synthesis’ procedure of the GO-based complexes from their ‘synthetic identity’ (grapholipoplexes) to their ‘biological identity’ (biocoronated grapholipoplexes). **b** Physical chemical characterization of DOTAP grapholipoploxes in terms of size and zeta potential changes by varying DOTAP/complex weight ratio (Rw) through DLS measurements. **c** Transfection efficiency (TE) measured as relative light units (RLU) to milligrams of proteins, and cell viability of GO, DOTAP and grapholipoplex once administered to HeLa cells. **d** Confocal microscopy images of Hela cells treated with DNA- red labeled GO complexes (left panel) and grapholipolexes (right panel). Cell nuclei are marked with DAPI. The same three characterization steps (i.e., DLS characterization, TE and cell viability and confocal microscopy experiments) were performed on multicomponent grapholipoplexes (panel **e**,** f**,and** g**, respectively) and multicomponent grapholipoplexes once incubated with different percentages of human plasma (HP) (panel** h**,** i**, and **j**, respectively). Sketch Adapted from Di Santo, et al. *Nanoscale* 11.6 (2019): 2733–2741; Di Santo, et al. *Applied Physics Letters* 114.23 (2019): 233,701 and Quagliarini, et al. *Pharmaceutics* 12.2 (2020): 113

The impressive progress made in gene therapies, such as gene silencing and editing has spurred efforts in identifying nucleic acid delivery vectors that are efficient, safe, and can be easily scaled up and produced consistently. To date, viral vectors have been the most popular option in gene-therapy clinical trials, outshining their non-viral counterparts in gene-transfer efficiency [24]. However, packaging restrictions and large-scale production constraints, in addition to the controversial safety profile, limited the introduction of viral vectors into clinics [25]. On the other hand, promising developments by non-viral carriers, mainly consisting of NPs of different sorts, circumvented some of such limitations [26]. Among these, 2D nanomaterials, including GO, have gathered considerable interest in biomedical applications thanks to their high surface-to-volume ratio, and ability to enhance cargo loading and transport [27]. Notably, GO is characterized by oxygen functional groups on its surface that allow for covalent and non-covalent functionalization, high aqueous dispersibility, and compatibility with biological environments [28], making it a building block for the fabrication of versatile functional nanomedicines. Despite these advantages, GO application in nucleic acid delivery is hindered by unfavorable electrostatic interactions resulting from negative charges in both vector and cargo. This is particularly relevant when double-stranded oligonucleotides are used, since the hydrophobic and π–π interactions between nucleobases and the GO lattice are stymied [29]. Previous studies have used GO to deliver double-stranded nucleic acids intracellularly [30], including plasmid DNA and small interfering RNA, but they relied on functionalizing the material with cationic polymers (e.g., polyethyleneimine (PEI), amine-functionalized dendrimers, polystyrene etc.,) [31–33],), polysaccharides (e.g., chitosan, starch, alginate, hyaluronic acid, and cellulose) [34, 35], or cell-penetrating peptides [36, 37] that have less-than-ideal biocompatibility. For instance, among cationic polymers, PEI suffers from the critical shortcoming of non-degradability that leads to severe cytotoxic effects [38]. Amine dendrimers interact with negatively charged cell membranes, disrupting their integrity and promoting cell apoptosis [39]. Studies also showed a correlation between cytotoxicity and dendrimer physicochemical properties. For example, the cytotoxicity of poly(amidoamine) (PAMAM) and poly(propylene imine) (PPI) dendrimers is directly proportional to concentration and the number of primary amine terminal zones [40]. Cationic polysaccharides, on the other hand, are hampered by their high dimension and potential immunogenicity. To surpass these limitations, one fascinating possibility involved coating GO sheets with lipids to create hybrid platforms. However, since the interaction between GO and lipid molecules is difficult to monitor, this strategy has always resulted challenging. In fact, hybrid platforms that include lipids are generally prepared either by breaking down their larger counterparts or assembling them from their building blocks. This later technique can be performed by a change in solvent polarity, temperature, or mixing of oppositely charged molecules. Liu et al. developed phospholipid-functionalized GO for drug delivery by reducing GO in the presence of anionic liposomes [40]. However, when anionic liposomes were replaced by cationic ones, the resulting composites aggregated in solution. Recent research by Frost et al. demonstrated that the interaction between GO and liposomes was strongly influenced by particle size [41]. If the liposome size is similar to or larger than that of the GO sheets, liposomes remain intact, and undesired aggregates form. When the size of the GO sheets is much larger (500 nm–5 μm) than that of the liposomes (200 nm), liposome rupture occurs, resulting in the decoration of the GO surface. Thus, it appeared clear that control over the size had to be a priority to guarantee efficient transfection. To this end, microfluidic devices provided ideal conditions for preparing hybrid nanosystems for gene delivery [42]. Microfluidics involves the manipulation of fluids in the microscale range. Under these conditions, minute volumes of fluids injected or pumped into the device are efficiently mixed under controlled flow conditions. We employed a microfluidic device to produce a hybrid gene delivery system made of GO nano-sheets surface-functionalized with the cationic lipid 1,2-dioleoyl-3-trimethylammonium-propane (DOTAP) and loaded with plasmid DNA [43] (Fig. 2a). The resulting gene delivery complexes, hereafter indicated as grapholipoplexes, were then validated through a multistep experimental strategy that involved (i) physical-chemical characterization in terms of size and surface charge through dynamic light scattering (DLS), (ii) biological validation through transfection efficiency (TE) and cell viability experiments, and (iii) cell internalization study through confocal microscopy. To ascertain the optimal ratio of DNA/grapholipoplex for cellular administration, we investigated the alterations in complex size and zeta potential by varying the weight ratio of DNA to grapholipoplex (Rw) (Fig. 2b). DOTAP grapholipoplexes exhibited typical features of lipoplexes such as charge inversion and re-entrant condensation as a function of the Rw [44]. Rw = 2 was chosen as combined low dimensions with negatively charged surface charge assuring complete surface coating with DNA. These optimized grapholipoplexes demonstrated remarkable efficiency in transfecting human cervical cancer cells (HeLa) while exhibiting minimal cytotoxicity when compared to pristine GO and DOTAP liposomes (Fig. 2c). To further interpret TE data, we explore HeLa uptake through confocal microscopy on both DNA-labeled GO and grapholipoplex (as shown in Fig. 2d). Hela cells treated with GO/DNA complexes contained just a few bright spots suggesting that the complexes were not efficiently internalized within cells. On the opposite, most of the cells treated with DOTAP grapholipolplex were found to be highly fluorescent-positive. This result aligned with TE findings and support the proof that grapholiplexes were more efficient in transfecting HeLa cells with respect to pristine GO.

A well-established concept in lipid-mediated gene delivery states that lipid mixtures are more fusogenic than single lipids [45, 46]. Incorporating very different lipid headgroups and/or aliphatic chains in lipid shells has been shown to generate asymmetric vesicles that enhance the biocompatibility and flexibility of conventional systems [47]. To take advantage of this, we decorated GO with lipid blends of cationic, and zwitterionic lipids [48]. The generated library of multicomponent grapholipoplexes was validated by the same multistep experimental strategy used for DOTAP grapholipoplexes (Fig. 2e). Since positively charged gene vectors can efficiently interact with cells by electrostatic attraction with negatively charged cell proteoglycans, here we selected both positively and negatively (Rw = 2) charged grapholipoplexes for the next biological validation. As expected, for each particle formulation, positively charged complexes were more efficient than their negatively charged counterpart. Furthermore, we noticed a significant impact of the lipid composition on the transfection efficiency (TE) of positively charged complexes. This led to TE values that varied by approximately one order of magnitude across different formulations. This finding aligns with the transfection behaviour commonly observed with cationic lipid-based systems. In fact, several studies have indicated that lipid composition plays a crucial role in determining the endosomal escape of lipid vesicles and the subsequent cytosolic release of the gene payload [49, 50]. Among positively charged grapholipoplex formulations (i.e., #4, #5, #6 and #8 in Fig. 2f), the grapholipoplex#8 (Rw = 0.2) made of DOTAP, (3β-(N-(N0,N0-dimethyl-aminoethane)-carbamoyl))-cholesterol DC-Chol and neutral cholesterol (Chol) (25%, 25%, and 50%, molar ratios respectively), resulted to be the best compromise between high TE and low cytotoxicity, even if compared with Lipofectamine 3000, the gold standard for lipid transfection. This can be attributed to the increased presence of cholesterol and cholesterol-like molecules that promote the formation of nonlamellar phases in the membranes of endosomes, thereby enhancing their propensity for endosomal escape [51]. Cellular uptake experiments performed on the worst and the best formulations (respectively #4 and #8) confirmed TE results (Fig. 2g). Approximately only 20% of HeLa cells treated with grapholipoplexes#4 showed positive fluorescence with a very limited number of cells engaged in DNA delivery, as represented by the complexes intracellular size distribution in the left panel. Conversely, when grapholipoplexes#8 complexes were administered to HeLa, these displayed approximately 90% of positive fluorescence cells with most of them arranged in the perinuclear region, as quantitively confirmed by the intracellular size distributions shown in the right panel. In summary, the hybrid platforms comprising lipid-covered GO have emerged as ideal candidates for gene transfection. These platforms demonstrate efficient gene condensation and protection, enhanced cellular uptake, controlled gene release, and high TE making them highly promising for gene delivery applications. In a more recent work, we asked whether the biomolecular corona of grapholipoplexes may have an impact on their TE and cytotoxicity [52]. To this end, we incubated the complexes with different percentages of HP, and we investigated the impact of protein concentration on their size and zeta potential (Fig. 2h). Biocoronated grapholipoplexes demonstrated a significant increase in size and a rapid transition of zeta potential from positive to negative values. As plasma proteins are predominantly anionic at physiological pH, even at a low protein concentration of 1% HP, the cationic surface charge of grapholipoplexes quickly shifted to negative values (zeta potential around − 20 mV). With increasing HP concentration, the zeta potential remained consistently negative with minimal fluctuations, indicating complete protein coverage of the complexes. Furthermore, a more complex size evolution pattern was observed. At 1% HP, the complexes exhibited larger sizes, indicating rapid particle clustering due to charge neutrality. As the HP concentration increased, there was a notable decrease in size until reaching a plateau of around 5% HP exposure. As a next step, biocoronated grapholiplexes were administrated to two breast cancer cell lines, i.e., MDA-MB and MCF-7 and one colorectal cancer cell line i.e., CACO-2 cells (Fig. 2i). TE exhibited a decreasing trend with increasing protein concentration, while a non-monotonic trend was observed for cell viability among the different conditions. Pristine grapholipoplexes reduced cell viability by up to 59.3%. On the other side, biocoronated grapholipoplexes increased cell viability up to 94.3% until HP = 10%vol. Further increase in protein concentration led to a further cell viability decrease. Our findings seemed to suggest that the interaction between the composition of the PC and the receptor profiles of cancer cells can influence the association between particles and cells, as well as the signalling of apoptosis-inducing ligands. While more in-depth research is necessary to confirm this suggestion, findings displayed in Fig. 2 are in accordance with previous studies [53, 54]. In general, the PC can have both detrimental and protective effects. On one hand, the PC may undergo denaturation and expose immunogenic epitopes, leading to a cytotoxic mechanism [55]. On the other hand, it can provide protection by creating a stealth effect that reduces the uptake of nanosystems by immune cells [56]. In addition, PC has also been shown to influence the intracellular localization of NPs [57]. Among the possible intracellular destinations, lysosomes are detrimental to gene vectors posing a significant obstacle to efficient transfection [58]. Therefore, we investigated the fate of fluorescently labelled grapholipoplexes. In Fig. 2j we reported confocal microscopy images of MDA-MB cells treated with fluorescently labelled pristine (left panel) and biocoronated grapholipoplexes (HP = 20%) (right panel). Lysosomal staining (red) was performed on the cells.

As a result, the colocalization of grapholipoplexes with lysosomes led to the formation of yellow clusters. Pristine grapholipoplexes demonstrated a favorable capacity to evade lysosomal degradation, while their coronated counterparts tended to accumulate within lysosomes. These findings align with the results obtained from TE experiments and support the hypothesis that the PC formed in a protein-rich environment, such as the physiological one, can impede the escape of gene delivery systems from endosomes. This, in turn, leads to their accumulation in lysosomal compartments, diminishing their effectiveness. However, recent research has demonstrated that pre-coating NPs with plasma proteins allows for the creation of artificial coronas with tailored physicochemical properties, enhancing transfection outcomes. According to these findings, biocoronated grapholipoplexes coated with artificial coronas formed at low protein concentration (HP < 2.5%) exhibited excellent TE while minimally affecting cell viability. This indicates that pre-coating grapholipoplexes could be a viable strategy to modulate their transfection behavior in vivo.

## Graphene oxide potential in drug delivery and cancer therapy: protein Corona Studies

**Fig. 3:**
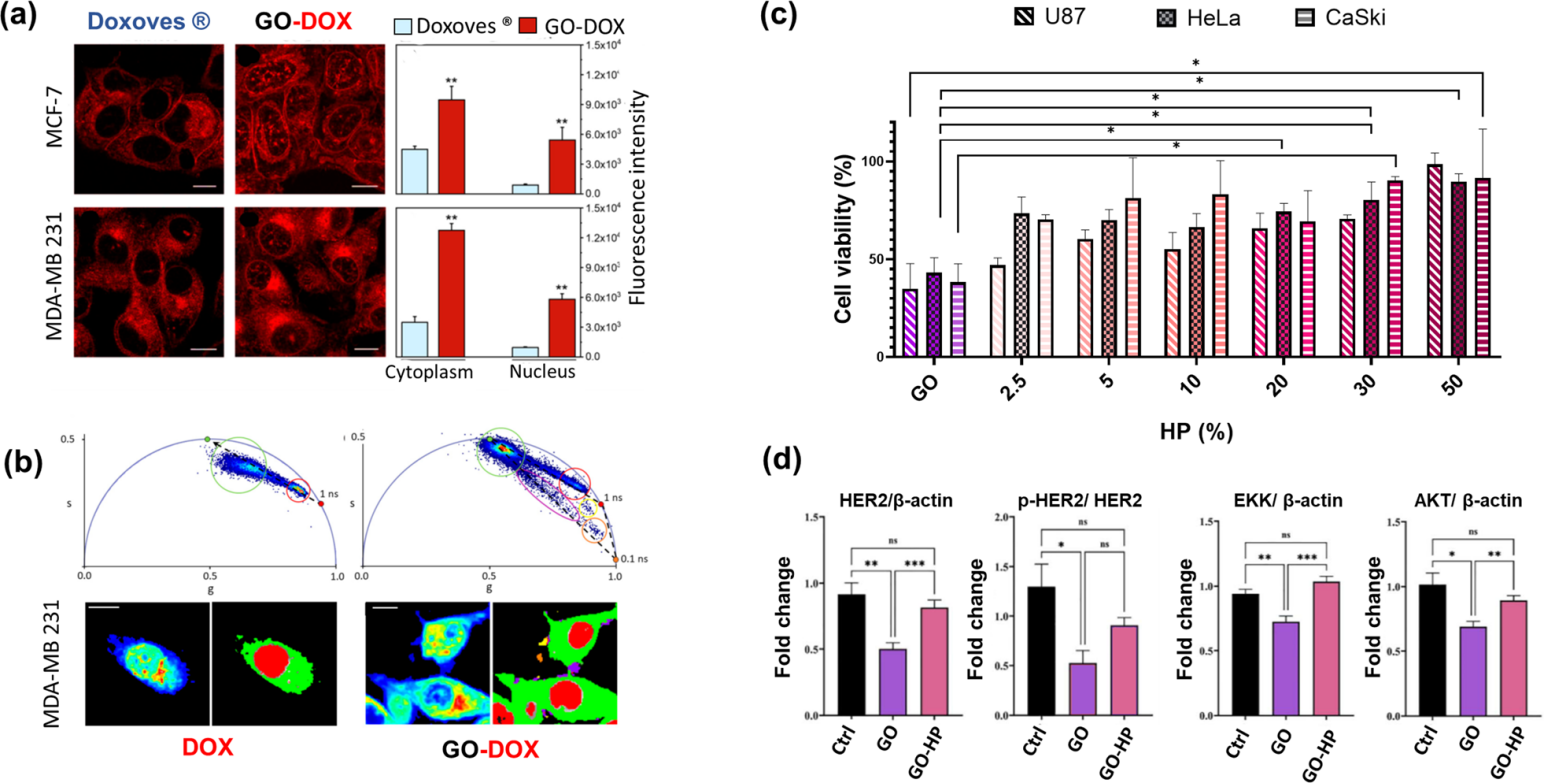
**a** Confocal microscopy images of MCF-F and MDA-MB 231 cells treated with commercial liposomal doxorubicin (DOX) Doxoves® and DOX-loaded graphene oxide (GO) formulation (GO-DOX). The hystogram plots show the fluorescence intensity of nuclear and cytoplasmic signals in cells related to Doxoves® and GO-DOX complexes. **b** Phasor fluorescence lifetime imaging microscopy (FLIM) analysis performed on MDA-MB 231 cells treated with free DOX (upper left panel) and GO-DOX (upper right panel). The phasor plots contain cluster of points corresponding to pixels with similar lifetime. The cluster are identified by specific region of interest (ROIs) related to each molecular species (e.g., free DOX with red ROI, DOX attached to biological membranes with green ROI etc.,). In bottom panels, intensity and lifetime images of DOX-treated cells and GO-DOX-treated cells coloured according to the ROIs. **c** Cell viability of U87, Hela and CasKi cells treated with naked GO and GO incubated with different percentages of human plasma (HP). **d** Densitometric quantification of HER-2, ERK, and AKT expression, normalized on β-actin, and of pHER-2/HER-2, from three independent experiments; one-way ANOVA test followed by Tukey’s multiple comparison test (*p < 0.05, **p < 0.01, ***p < 0.001). Adapted from Quagliarini et al., *Nanomaterials* 10.8 (2020): 1482. and Cui et al. *Nanoscale Advances* 4.18 (2022): 4009–4015

GO has attracted increasing interest in the fields of drug delivery and cancer therapy owing to its planar and π-conjugated structure, which endows it with an excellent ability to immobilize substances such as metals, drugs, biomolecules [59–61]. Additionally, the high concentration of reactive oxygen groups on GO surface enhances its functionalization ability with polar polymers or polar molecules, making it an excellent candidate for GO/polymer composites [62, 63]. These active groups are also perfect for immobilizing molecules on the GO surface, making it hydrophilic and an excellent choice for the delivery of drugs. D. Ananya and R. Vimala developed a unique drug delivery system made of chitosan polymerised GO to attain an anticancer drug delivery towards MCF-7 breast cancer cells [64]. Among functionalization methods utilized to improve GO properties, PEGylation (PEG-polyethylene glycol), resulted in the most suitable since proved enhanced biocompatibility, solubility, and stability of GO in physiological conditions. As an instance, the use of PEG-functionalized GO as a nanocarrier to bind water-insoluble anticancer drugs was evaluated for its cytotoxicity towards human colon cancer cells by Z. Liu et al., [65]. If the comparison is extended to conventional delivery systems, such as lipid-based systems, graphene-based nanomaterials in several cases proved to be more efficient for drug loading and delivery [4]. As instance, in our previous study, we demonstrated the superior efficiency of GO in delivering the anticancer drug doxorubicin (DOX) respect to a commercially approved DOX-loaded liposomal formulation (Doxoves®), whose use has raised numerous controversies for the potential toxicity at high dosages [66]. DOX exerts its therapeutic effects by intercalating into nuclear DNA. Consequently, to maximize the anticancer efficacy of DOX, the drug must be efficiently internalized by cancer cells and subsequently delivered to the cell nucleus. To investigate the intracellular distribution of DOX in cancer cells, we employed confocal microscopy. Figure 3a displays representative confocal images of two breast cancer cells, i.e., MCF-7 and MDA-MB-231 cells, treated with Doxoves^®^ and GO-DOX complexes. The quantitative analysis of nuclear and cytoplasmic signals presented in the histogram plots shows that the nuclear fluorescence in cells treated with GO-DOX complexes was about five times higher than that observed in cells treated with Doxoves® for both cell lines. To get insights into the intracellular and intranuclear DOX behaviour, we conducted fluorescence lifetime imaging microscopy (FLIM) on cells treated with GO-DOX, using the free drug as a control (Fig. 3b). FLIM can distinguish free DOX from DOX adsorbed/attached to GO. In the upper panels, the FLIM analysis is presented as a phasor representation of lifetimes measured in cells exposed respectively to free DOX (left panels) and GO-DOX (right panels). The phasor plot displays clusters of data points representing pixels with similar lifetime spectra. These clusters can be identified and isolated using specific regions of interest (ROI). In the left panels, green ROI and red ROI identify the areas with pixels related to DOX in the cytoplasm and the nucleus respectively. In the right panels, violet, orange, and yellow clusters identify ROIs related to the naked carrier (GO) and the released drug (both free or associated with cellular membranes). These findings collectively emphasize the presence of specific micrometric patches along the cell border, as better illustrated in the lower right panels. We attributed these patches to the areas where GO-DOX complexes are adhering to the cell membrane and eventually releasing the drug. Our data are in agreement with previous evidence indicating that GO likely binds to integrins at the cancer cell’s plasma membrane, activating the integrin-FAK-Rho-ROCK pathway and rendering cancer cells more susceptible to chemotherapeutic agents [67]. To harness the full potential of GO, it is imperative to gain a comprehensive understanding of the mechanisms that govern GO-cell interactions. By unravelling these intricate mechanisms, we can pave the way for innovative strategies and drive advancements in the market of nanoparticle-based therapies for cancer treatment [68]. However, successful incorporation of GO into cancer therapeutics requires a comprehensive understanding of the interface between GO itself and the biological environment [69]. Motivated by the necessity of developing reliable GO-based anticancer therapeutics, we validated the anticancer capacity of GO in both its synthetic and biological forms and we got insights into the molecular mechanisms underlying the GO anticancer potential [70]. We found that exposing GO to increasing percentages of HP resulted in a high impact on the GO anticancer activity with a marked increase of cell viability in three different models of cancer cell lines i.e., U-87 human glioblastoma multiforme cell line, HeLa cell line, and CasKi human cervical epidermoid carcinoma cell line, with respect to naked GO (Fig. 3c). This suggested that in a protein-enriched physiological environment, the anti-cancer effect of GO may be impaired probably due to a reduction in cell penetration. To validate this hypothesis, we further studied the impact of naked GO and GO incubated with a high percentage of HP on human epidermal growth factor receptor 2 (HER-2) expression in SK-BR-3 human breast cancer cells, a model system of HER-2 positive cancer cells. A western blot analysis on treated SK-BR-3 showed that GO treatment led to a significant reduction in overall HER-2 levels, accompanied by down regulation of expression and activation of HER-2-driven signalling pathways such as phosphatidylinositol-3-kinase (PI3K)/proteinkinase B (AKT) and mitogen-activated protein kinase (MAPK)/extracellular signal-regulated kinase (ERK) pathways, which mediate cancer cell survival and proliferation. However, PC reversed the impact of GO on HER-2 expression and its downstream molecular effects, bringing them back to the control level (Fig. 3d). These results demonstrated that PC overrides GO anticancer ability by interdicting GO physical interaction with HER-2 exposed to cell membranes. In conclusion, PC plays a significant role in modulating the behaviour and efficacy of nanocarriers. Understanding the interactions between nanocarriers and the PC is essential for harnessing their full potential in clinical translation. Further studies are needed to explore and optimize the bio-nano interactions, considering the complex biological environment, to pave the way for advanced nanomedicine design and improved cancer therapies.

## Interrogating the personalized protein Corona of Graphene Oxide: a new approach for early disease detection

**Fig. 4:**
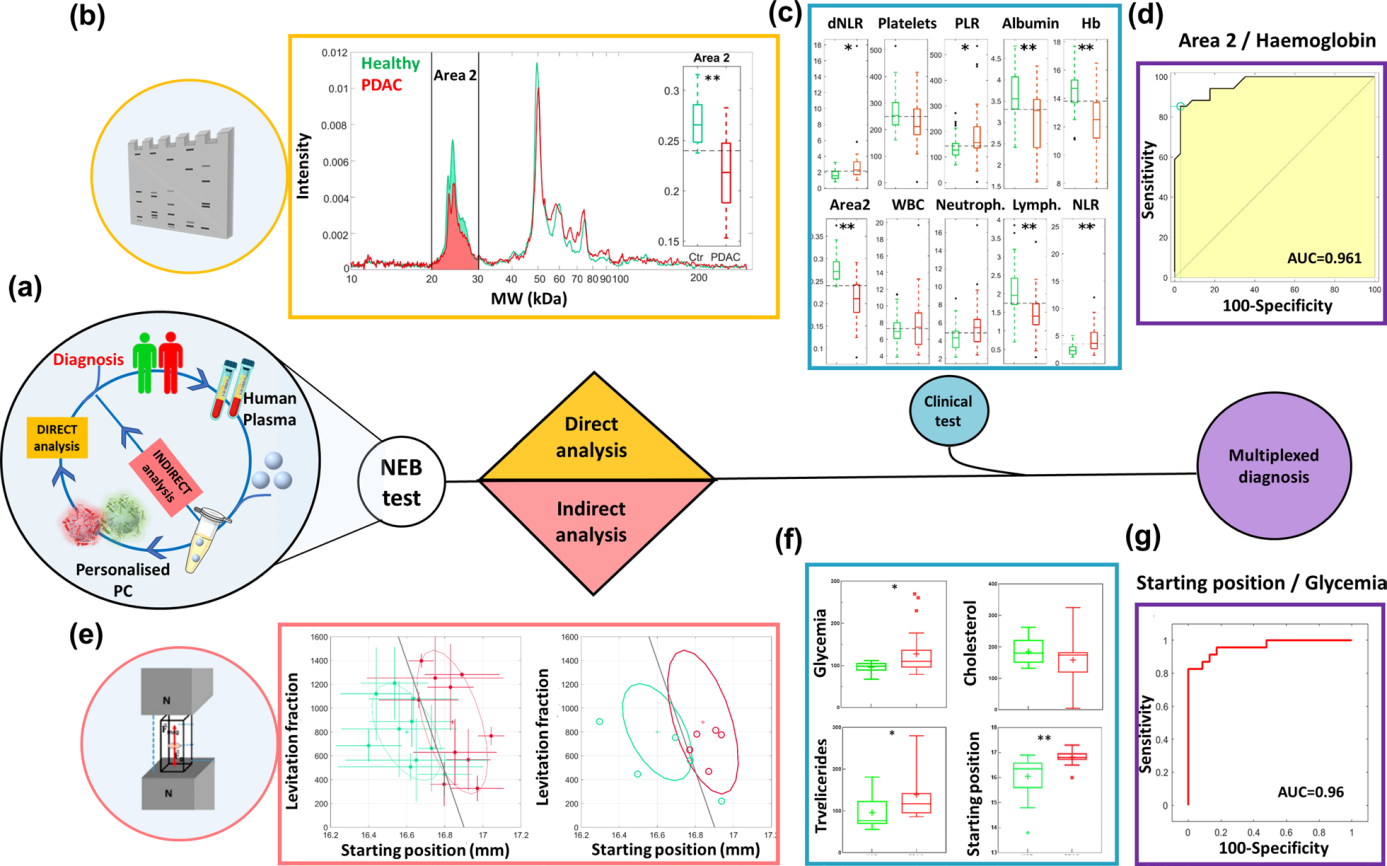
**a** Schematic workflow of nanoparticle-enabled blood (NEB) test for cancer detection. Human plasma is collected from healthy and oncological individuals and incubated with nanoparticles (NPs) to generate personalised NP-protein coronas (PCs) complexes further characterised by direct or indirect analysis. The PC characterization readouts can be paired with clinical blood levels to enhance the diagnostic power of the test. **b** 1D profiles obtained by SDS-PAGE images derived from direct analysis of personalised graphene oxide (GO)-PCs related to 34 healthy (green) and 34 oncological (red) individuals. Black lines identify the most discriminant molecular weight (MW) region between 20–30 kDa (Area 2). Boxplot of the computed Area 2 for all the processed samples is reported in the inset. ** indicate a Student p-value < 0.001. **c** Box plots of electrophoretic and clinical blood levels for oncological (red) and healthy (green) sample distributions. Asterisks correspond to Student *p*-values: * *p* < 0.05; ** *p* < 0.001. **d** AUC obtained by coupling Area 2 and haemoglobin (Hb) as classifiers. **e** Scatter plot of the Maglev signatures derived from indirect analysis of personalized NP-PCs complexes from 10 healthy and 10 oncological subjects. The black line is the output of linear discriminant analysis (left panel). The output of a blind validation test performed on 5 healthy and 5 oncological samples and superimposed with the distribution of the training test (ellipses) (right panel). **f** Distributions of Maglev fingerprint and blood levels of 22 healthy and 24 oncological subjects **g** Receiving operating curve and AUC calculated from the coupling between glycemia blood level and Maglev starting position of the 22 healthy and 24 oncological subjects. Figure adapted from Caputo, D. et al., *Cancers* 13.1 (2020): 93.; Digiacomo, L. et al. *Cancers* 13.20 (2021): 5155. and Quagliarini, E. et al. *Cancer Nanotechnology* 14.1 (2023): 1–12

Numerous investigations have elucidated that the protein patterns bound to nanosystems are not mere representations of the human proteome composition [71]. In fact, only a few dozen plasma proteins, accounting for approximately 99% of the total plasma volume, are typically present on the surface of nanosystems. Conversely, nanomaterials serve as effective protein accumulators, exhibiting a distinctive affinity and a low dissociation rate for proteins [72]. Recent studies have highlighted that a protein with low abundance in the plasma can become one of the most abundant proteins in the PC around a nanosystem [73, 74]. These discoveries have introduced the concept of “personalized PC,“ wherein the composition is influenced by changes in the concentration and structure of individual plasma proteins in each patient [75, 76]. In other words, when nanoparticles are incubated with plasma from patients with different pathologies, distinct PCs may form. Several diseases, including cancer, are associated with alterations in the patients’ proteome, leading to significant changes in the identity of PCs. The discovery of personalized PCs has revolutionized the field of nanomedicine, expanding its applications to tumour diagnosis and prognosis. Currently, most techniques for PC analysis rely on proteomics, with mass spectrometry (MS) being fundamental in most of the proposed experiments [77]. The exceptional sensitivity of MS enables the detection of subtle changes in the human proteome, allowing the identification of individual protein biomarkers and providing information about the composition and function of PCs. However, these approaches have limitations due to their labour-intensive and costly procedures, making them unsuitable for large-scale production. The World Health Organization (WHO) emphasizes that cancer screening and detection procedures must meet the REASSURED (Affordable, Sensitive, Specific, User-friendly, Rapid and robust, Equipment-free, and Deliverable to end-users) criteria [78]. Therefore, researchers are exploring the integration of low-resolution benchtop techniques to develop cost-effective and efficient screening procedures. In this regard, nanoparticle-enabled blood (NEB) tests have emerged as a rapid and economical technology for characterizing PCs in early cancer detection [79–81]. NEB tests involve the evaluation of NP-PC characteristics, such as size, surface charge, and composition, using simple techniques like DLS, microelectrophoresis (ME), and one-dimensional sodium dodecyl sulfate-polyacrylamide gel electrophoresis (1D-SDS-PAGE). For instance, by incubating NPs with biological fluid from healthy individuals and those affected by cancer, information about the clinical status of subjects can be obtained by analysing the upregulation or downregulation of corona proteins within specific molecular weight (MW) ranges of the SDS PAGE profile [82]. Compared to conventional proteomic techniques such as MS, the key advantage of NEB tests lies in their ability to provide a comprehensive evaluation of the protein pattern. This allows the differentiation between donor groups based on systematic alterations in multiple proteins, considering changes in NPs, tumour stage, or cancer type. Typically, NEB tests are performed in a step-by-step workflow as schematically represented in Fig. 4a. These steps include (i) the collection of clinically relevant body fluids from healthy and oncological subjects. To date, only serum and plasma have been used, while other fluids are currently under investigation; (ii) the synthesis of a library of NPs with different physical-chemical properties, (iii) the choice of exposure conditions between NPs and body fluids to generate nanoparticle-protein complexes, (iv) the analysis of protein composition of the complexes, and (v) the statistical study of experimental data to obtain the final diagnosis. The test structure has many degrees of freedom that can affect its prediction ability including the physical-chemical properties of NPs, the exposure conditions such as protein concentration, shear stress, exposure time, and temperature or the biological source (e.g., plasma, serum, saliva etc.,) [83]. Among these, the detection technique for PC analysis may include two different methodological approaches, i.e., direct analysis of the PC isolated from the nanoparticle surface and indirect analysis of the PC which consists of an *in-situ* evaluation of the NP-PC complexes. Finally, the outcomes of NEB tests can be further paired to clinically relevant parameters in multiplexed strategies, to improve the classification ability of the test. As an illustrative example, the combination of blood levels of haemoglobin (Hb), albumin, lymphocyte, and platelet has emerged as a paramount prognostic factor for postoperative survival among patients diagnosed with pancreatic ductal adenocarcinoma (PDAC) [84]. Additionally, systemic inflammatory response biomarkers (SIRBs), including white blood count (WBC), neutrophils to lymphocytes ratio (NLR), derived-NLR (d-NLR), and platelets to lymphocytes ratio (PLR), have garnered significant attention in the realm of tumor diagnosis and prognosis [85]. Consequently, the vast amount of information amassed by medical and laboratory teams can be systematically evaluated and interlinked to yield a highly accurate diagnostic test. In line with this notion, a considerable portion of our recent research efforts has been dedicated to developing multiplexed tests that intricately integrate clinical biomarkers with the readouts obtained from NEB tests [86]. Among nanomaterials selected for our NEB tests, GO emerged for its low-cost production, high dispersibility in water solvents, and the presence of reactive oxygen groups on its surface. Additionally, GO lower affinity toward albumin, the most abundant blood protein, allows for preferential bonding with proteins present at lower concentrations in blood, enhancing the sensitivity of differentiation between different protein classes [87]. In one of our works, we adopted a multiplexed GO-based blood test that paired the outcomes from SDS-PAGE profile, performed on personalised PC derived from healthy and PDAC affected donors, with clinical biomarkers such as Hb, lymphocyte, WBC, NLR, d-NLR, and PLR [88]. 1D-SDS-PAGE is particularly suitable for distinguishing protein patterns within NEB tests, as it offers qualitative outcomes that enable simultaneous resolution and distinction of various protein coronas resulting from different NP incubation conditions [89]. We observed that the judicious fusion of low-molecular-weight proteins between 20 and 30 kDa (referred to as Area 2 in Fig. 4b) with Hb blood levels (Fig. 4c) resulted in an area under the curve (AUC) of 0.961, thus overcoming the prediction ability of a single parameter (Fig. 4d). Over ten years, our research has conclusively demonstrated that NEB tests serve as powerful tools for early cancer detection and hold the potential to catalyse the development of innovative technologies for the discovery of new biomarkers. Nonetheless, it is important to acknowledge that this technology is not exempt from limitations. Among the challenges faced, the isolation of PC necessitates a multitude of intricate steps, which, in turn, may introduce inter-operator variability, thereby compromising the reliability of the obtained results. To address this concern, indirect methods for PC characterization have gained prominence in recent years as promising alternatives to streamline the experimental steps without compromising the effectiveness of the test, while concurrently enhancing reproducibility, especially when dealing with extensive datasets [79]. Indirect approaches for PC characterization involve examining the NP-protein complex as a cohesive entity, enabling the extraction of valuable information about its size, shape, surface charge, nanostructure, and mass. Techniques such as DLS, ME, and fluorescence lifetime analysis have proven to be invaluable in this regard. Notably, the employment of magnetic levitation (Maglev) has emerged as a robust technique for the indirect characterization of NP-protein complexes [90, 91]. This methodology leverages the application of an intense magnetic field to differentially separate objects [92]. When a diamagnetic NP is injected in the test cuvette of a MagLev device it can levitate and equilibrate at different heights depending on the intensity of magnetic field gradient, exposure time and, most importantly, on the particle density. Since personalised PCs have different compositions and densities the levitating profiles along the magnetic field gradient can be used to distinguish healthy from oncological donors. In several of our recent studies, we harnessed the power of Maglev to characterize GO-PCs originating from both healthy subjects and oncological individuals affected by various types of cancer [93]. Among different Maglev signatures, the ‘starting position’ of the PC-NP complexes i.e., the position reached when the complexes were exposed to the magnetic field, and the area of the levitating fraction of the sample at the equilibrium state (referred to as ‘levitating fraction area’) were identified as the most discriminant Maglev signatures to distinguish healthy from oncological subjects. Particularly, as shown in the left scatterplot of Fig. 4e, linear discriminant analysis (LDA) performed by coupling Maglev starting position and levitating fraction area of corona-coated GO complexes derived from 10 healthy and 10 PDAC-affected individuals, allowed high discrimination between the two classes, with only two PDAC subjects misclassified, meaning a specificity of 80%, sensitivity of 100%, and overall classification accuracy of 90%. To validate the aforementioned classification by MagLev fingerprints, a blind validation test was also performed on a cohort of 5 healthy and 5 PDAC samples. As shown in the right panel, only one healthy sample was misclassified by the test, which thus reached a global accuracy value of 90%. Finally, since we demonstrated that a proper combination of non-specific laboratory data (e.g., low Hb levels), with the outcomes of GO-based NEB tests, discriminated PDAC patients from healthy controls with high diagnostic accuracy, in a recent work we assessed the ability of the MagLev test in detecting PDAC when coupled with the blood levels of glycemia, cholesterol, and triglycerides (Fig. 4f) [94]. The multiplexed strategy was validated using a sample cohort made of 24 PDAC patients and 22 healthy volunteers and its most optimised version was obtained by coupling the starting position with the patients’ glycemia levels, obtaining an AUC of 0.96 (Fig. 4g). Although still in the exploratory phase, the potential implications of this technology, if substantiated on a large cohort, are poised to revolutionize clinical practice by enabling rapid and robust cancer detection methodologies.

## Conclusions

In summary, a glimmer of opportunity is opening in the development of clinically applicable theranostic solutions thanks to the exploitation of GO in the biomedical field. Passing from gene delivery to drug delivery and diagnostics, GO seems to provide interesting new alternatives for the development of highly-performing vectors for nucleic acids, drugs, and biomolecules, in many cases surpassing the technologies already on the market in terms of biocompatibility, reproducibility and costs. Notably, GO also holds great promise in the fields of gene therapy and drug delivery for cancer treatment. Efforts have been made to improve the efficiency and safety of nucleic acid delivery vectors, with GO emerging as a valuable non-viral option. Functionalizing GO with lipids has been explored to enhance its gene delivery capabilities. Microfluidic devices have been used to monitor GO-based hybrid gene vectors which have demonstrated efficient gene transfection with low cytotoxicity. In drug delivery, GO’s planar structure and functionalization abilities have made it suitable for loading and delivering drugs. It has shown advantages over conventional lipid-based systems in terms of drug loading and stability showing superior efficacy in delivering anticancer drugs compared to approved formulations. Based on the collective experimental findings presented in this review, it can be inferred that PC has a substantial impact on various interactions involving GO. PC exerts inhibitory effects on the cytotoxicity induced by GO on tumor cells or influences immune response activity and biodistribution. Given the challenges associated with precisely controlling protein interactions in vivo, many strategies aimed at modulating the PC rely on functionalization with artificial corona that suppress protein adsorption and reduce lysosomal escape. The unique properties of biocoronated GO hold potential for specific cell targeting applications. Although the compositions of GO corona are still being studied, initial data are encouraging. For example, the enrichment of ApoE residues in the graphene-based materials corona could facilitate the traversal of the blood-brain barrier and enable targeting of the cerebrovascular endothelium for the treatment of neurological diseases [95]. Moreover, when immersed in plasma from oncological patients, GO-PC exhibits unique characteristics that can be exploited to develop PC-based diagnostic methods.

In this review, we summarized and critically discussed the main achievements regarding the use of GO in biomedical applications over the past decade. The upcoming one is expected to definitively bring GO technologies from basic research to clinical practice. Notably, the arising concept of PC in addition to revolutionizing most nanotechnologies, will bring new opportunities, especially for graphene-based materials. In conclusion, we expect that the achievements thus far represent just the beginning of a long journey towards new fascinating applications of graphene-based materials in theranostics.

## Data Availability

The data that support the findings of this study are available from the authors upon reasonable request.
